# Effects of added exogenous hormones on lactation-related physiological functions of equine mammary epithelial cells

**DOI:** 10.3389/fvets.2025.1660502

**Published:** 2025-11-18

**Authors:** Chao Li, Shengchen Zheng, Jianwei Lin, Qian Li, Kailun Yang, Xiaobin Li

**Affiliations:** Xinjiang Herbivore Nutrition Laboratory for Meat & Milk, College of Animal Science, Xinjiang Agricultural University, Urumqi, China

**Keywords:** equine mammary epithelial cells, hydrocortisone, epidermal growth factor, cell viability, transcriptome

## Abstract

This study aimed to optimize the culture conditions for equine mammary epithelial cells (EMECs) by investigating the effects of fetal bovine serum (FBS), hydrocortisone (HYD), insulin (INS), and epidermal growth factor (EGF) on cell growth and function. The primary objectives were to identify the optimal culture conditions for EMECs, evaluate the impact of FBS, HYD, INS, and EGF on cell viability and milk component synthesis, and uncover genes involved in mare lactation and milk production. The optimal FBS concentration for cell survival was determined to be 15%, with further improvements achieved through the individual addition of 1 μg/mL HYD, 5 μg/mL INS, and 5 ng/mL EGF. Orthogonal analysis revealed that the combination of 1 μg/mL HYD and 5 ng/mL EGF resulted in the highest survival rates. This combination significantly increased triglyceride and lactose production by 86.36% and 33.33%, respectively (*P* < 0.01), and β-casein levels by 30.10% (*P* < 0.05), compared to the control. Transcriptome sequencing identified 596 upregulated and 432 downregulated genes, including *laminin subunits* (*LAMA2, LAMA3, LAMA4, LAMA5*), *laminin gamma-1* (*LAMC1*), *collagen type IV* α*1 chain* (*COL4A1*), *fatty acid synthase* (*FASN*), and *forkhead box protein O1* (*FOXO1*). Functional enrichment analysis highlighted key pathways related to cell adhesion, bioadhesion, cell-cell signaling, ECM-receptor interactions, and the PI3K-Akt signaling pathway. These findings demonstrate that the addition of HYD, INS, and EGF, both individually and in combination, enhances EMEC viability and milk component synthesis, offering new insights into the molecular mechanisms of mare lactation and milk production.

## 1 Introduction

With the increasing recognition of mare's milk as a functional food, its nutritional and biological properties have gained significant attention. Compared to cow's milk, mare's milk is distinguished by its higher levels of polyunsaturated fatty acids, an abundance of vitamins, and lower cholesterol content (1). Additionally, mare's milk demonstrates a variety of bioactive effects, including antiviral, anti-inflammatory, and antifatigue properties, and is believed to offer therapeutic benefits for conditions such as hyperlipidemia and diabetes (2). Consequently, a deeper understanding of the physiological mechanisms driving lactation in mares is crucial for enhancing milk yield and quality, as well as for facilitating its application in the development of functional foods and health-related products.

Research on mare lactation has predominantly focused on genetic selection and dietary interventions to optimize lactation characteristics. However, the biological regulatory mechanisms, particularly at the cellular level, such as the proliferation of mammary epithelial cells (MECs) and the synthesis of key milk components, remain insufficiently explored. MECs are pivotal functional units in lactation, and their responsiveness to hormonal signals plays a critical role in the synthesis of milk fat, lactose, and proteins (3). In dairy cows and goats, hormones such as hydrocortisone (HYD), insulin (INS), and epidermal growth factor (EGF) have been shown to regulate mammary gland development and milk composition. Specifically, HYD is known to activate secretory functions, INS facilitates lipogenesis, and EGF, which is upregulated during lactation, helps sustain secretory activity (4–6).

However, the effects and underlying molecular mechanisms of these hormones in mare MECs remain largely unexplored. The relevant signaling pathways and their downstream target genes are yet to be fully defined. This study aims to establish an *in vitro* culture system for mare MECs and systematically assess the impact of HYD, INS, and EGF on cell proliferation and the synthesis of milk fat, lactose, and milk proteins. Additionally, transcriptomic techniques will be applied to identify candidate genes associated with lactation and milk component biosynthesis in mares. These insights are expected to provide a deeper understanding of the molecular mechanisms of mare lactation and contribute to improvements in milk quality and lactational performance.

## 2 Materials and methods

### 2.1 Experimental cell source and culture

The third-generation equine mammary epithelial cells (EMECs) used in this study were provided by the Laboratory of Meat Dairy Herbivore Nutrition, College of Animal Science, Xinjiang Agricultural University (7). Mammary tissue was obtained from lactating Kazakh mares via surgical excision. The tissue was then digested using an enzymatic method with 0.1% Type I collagenase and 0.01% hyaluronidase to extract primary cells. The cells were seeded into culture flasks and cultured at 37 °C with 5% CO_2_. During the culture process, fibroblasts were removed by trypsinization, and multiple rounds of purification were performed to obtain a relatively pure population of mammary epithelial cells (MECs). Morphological examination revealed that the cells exhibited typical elongated or island-like arrangements and displayed a characteristic “S”-shaped growth curve. Agarose gel electrophoresis confirmed the expression of cytokeratin 18 (CK18), a marker for mammary epithelial cells, and the absence of vimentin (VIM), a fibroblast marker. Further immunofluorescence staining confirmed the positive expression of CK18, thereby validating the cells as mammary epithelial cells (EMECs).Furthermore, transcriptomic or gene expression analyses in this study confirmed the absence of fibroblast markers (vimentin, collagen, α-SMA, fibronectin, etc.), further verifying that the cells are equine mammary epithelial cells.

### 2.2 Hormone sources and concentration selection rationale

The hormones used in this experiment were hydrocortisone (HYD, B21001, Shanghai Source Leaf Biotechnology Co., Ltd., China), insulin (INS, I8830, Beijing Solebao Technology Co., Ltd., China), and epidermal growth factor (EGF, P02835, Beijing Solebao Technology Co., Ltd., China), all of which are commercially available products. The selection of hormone concentrations was based on previous studies, including those by Spaas et al. (8), Bartlett et al. (9), and Ledet et al. (10). Spaas et al. used 10 μg/mL of insulin to support the viability of equine mammary stem cells; Bartlett et al. maintained equine mammary organoids in a medium containing 5 μg/mL of insulin, 1 μg/mL of hydrocortisone, and 1 ng/mL of EGF, which effectively supported their secretory activity; Ledet et al. applied similar concentrations to study hormone-responsive signaling pathways. Based on these studies, we tested a range of insulin concentrations (1–10 μg/mL), hydrocortisone concentrations (0.1–5 μg/mL), and EGF concentrations (1–10 ng/mL) in single-factor experiments to determine the optimal concentrations for promoting cell proliferation and lactation-related functions. These concentrations were then used in subsequent combined treatments and transcriptomic analyses.

### 2.3 Preparation of solutions

All solutions and reagents were prepared following standard laboratory procedures. These include 1% PBS solution, DMEM/F12 medium supplemented with 10%, 15%, and 20% FBS, as well as stock solutions of HYD, INS, and EGF. All experimental culture media were prepared in 50 mL volumes. Except for the FBS concentration screening experiment, each 50 mL of DMEM/F12 medium was supplemented with 500 μL of penicillin-streptomycin solution and 7.5 mL of fetal bovine serum (FBS). Following the conditions outlined in Tables 1, 2, different doses of HYD, INS, and EGF were added to the medium to obtain various hormone concentrations. All solutions were filtered through a 0.22 μm filter membrane and stored at appropriate temperatures. Detailed preparation methods and storage conditions are provided in Supplementary material.

**Table 1:** Addition of hormone master batch in one-factor test medium (μL).

**Items**	**Considerations**		
	**HYD**	**INS**	**EGF**
1 μg/mL HYD	50	0	0
5 μg/mL HYD	250	0	0
5 μg/mL INS	0	100	0
10 μg/mL INS	0	200	0
5 ng/mL EGF	0	0	125
10 ng/mL EGF	0	0	250

Stock solutions were prepared as follows: HYD (20 mg dissolved in 20 mL anhydrous ethanol, 1 mg/mL), INS (20 mg dissolved in 8 mL 0.01 mmol/L HCl, 2.5 mg/mL), and EGF (20 μg dissolved in 10 mL PBS, 2 μg/mL). All solutions were filtered through a 0.22 μm membrane and stored at −20 °C. The volumes listed in the table represent the amounts of stock solutions added to the one-factor test medium to achieve the target working concentrations.

**Table 2:** Addition of hormone mother liquor in orthogonal test medium (μL).

**Items**	**Considerations**		
	**HYD**	**INS**	**EGF**
Test group I	0	0	0
Test group II	0	100	125
Test group III	0	200	250
Test group IV	50	0	125
Test group V	50	100	250
Test group VI	50	200	0
Test group VII	250	0	250
Test group VIII	250	100	0
Test group IX	250	200	125

### 2.4 One-way experiment

First, different FBS concentrations (10%, 15%, 20%) were used to treat equine mammary epithelial cells (EMECs), and cell viability was measured to screen for the optimal serum concentration. Subsequently, under the optimized FBS concentration condition, different concentrations of hydrocortisone (HYD, 0, 1, 5 μg/mL), insulin (INS, 0, 5, 10 μg/mL), and epidermal growth factor (EGF, 0, 5, 10 ng/mL) were added to evaluate the individual effects of these hormones on cell viability.

#### 2.4.1 FBS concentration screening

Under the 10% FBS condition (with a blank control group), EMECs were seeded into 96-well plates at a density of 3 × 10^3^ cells/100 μL, with 100 μL per well and three replicates per group. The plate was incubated at 37 °C with 5% CO_2_ for 24 h. After 24 h, the medium was discarded, and the cells were washed twice with 1% PBS. Then, culture medium containing 10%, 15%, and 20% FBS was added, and the cells were incubated at 37 °C with 5% CO_2_ for 72 h. After 72 h, the medium was discarded again, and the cells were washed twice with 1% PBS. Subsequently, culture medium with 10%, 15%, and 20% FBS was added along with 10 μL of CCK-8 reagent, and the cells were incubated at 37 °C with 5% CO_2_ for an additional 4 h. After this incubation period, the optical density (OD) value was measured at 450 nm. Cell viability was calculated as follows:

Cell viability = (OD of experimental group – OD of blank group)/(OD of control group - OD of blank group)

#### 2.4.2 Hormone concentration screening

Based on the FBS concentration screening results, 15% FBS was selected as the optimal concentration. EMECs were seeded into 96-well plates at a density of 3 × 103 cells/100 μL, with 100 μL per well, and three replicates per group. The cells were incubated at 37 °C with 5% CO_2_ for 24 h. After 24 h, the medium was discarded, and the cells were washed twice with 1% PBS. Then, different concentrations of HYD (0, 1, 5 μg/mL), INS (0, 5, 10 μg/mL), and EGF (0, 5, 10 ng/mL) treatment solutions were added, and the cells were incubated at 37 °C with 5% CO_2_ for 72 h. After 72 h, the medium was discarded again, and the cells were washed twice with 1% PBS. The same concentration of hormone-containing medium was replenished, and the cells were incubated for an additional 72 h. At the end of the final incubation, the cells were washed twice with 1% PBS, and 100 μL of the corresponding medium was added. Then, 10 μL of CCK-8 reagent was introduced, and the cells were incubated at 37 °C with 5% CO_2_ for another 4 h. After this period, the OD value was measured at 450 nm.

### 2.5 Orthogonal experiment

An orthogonal experiment with three factors and three levels was designed using the L9 (11) orthogonal table to assess the effects of mixed additions of hydrocortisone (HYD, 0, 1, 5 μg/mL), insulin (INS, 0, 5, 10 μg/mL), and epidermal growth factor (EGF, 0, 5, 10 ng/mL) on the viability of equine mammary epithelial cells (EMECs) and to identify the optimal culture conditions. The experiment was conducted under 15% FBS medium (with a blank control group). EMECs were seeded into 96-well plates at a density of 3 × 10^3^ cells/100 μL, with 100 μL per well and three replicates per group. The cells were incubated for 24 h. After incubation, the medium was discarded, and the cells were washed twice with 1% PBS. Then, culture media containing different hormone combinations were added to each well, and the cells were incubated for another 24 h. After 24 h, the medium was discarded again, and the cells were washed twice with 1% PBS. Subsequently, 100 μL of the corresponding hormone-containing medium and 10 μL of CCK-8 reagent were added, and the cells were incubated for an additional 4 h. After this incubation, the optical density (OD) value was measured at 450 nm using a microplate reader. The specific factor levels and experimental groupings are detailed in Tables 3, 4.

**Table 3:** Orthogonal test factor level table.

**Items**	**Considerations**		
	**HYD**, μ**g/mL**	**INS**, μ**g/mL**	**EGF, ng/mL**
1	0	0	0
2	1	5	5
3	5	10	10

**Table 4:** Orthogonal test grouping table.

**Items**	**Considerations**		
	**HYD**, μ**g/mL**	**INS**, μ**g/mL**	**EGF, ng/mL**
Test group I	0	0	0
Test group II	0	5	5
Test group III	0	10	10
Test group IV	1	0	5
Test group V	1	5	10
Test group VI	1	10	0
Test group VII	5	0	10
Test group VIII	5	5	0
Test group IX	5	10	5

Test groups I–IX represent different combinations of hydrocortisone (HYD), insulin (INS), and epidermal growth factor (EGF) concentrations based on an orthogonal experimental design. Each group corresponds to a unique treatment condition as shown in the table. Cell viability was measured under each condition.

The results of the orthogonal experiment were analyzed by comparing the OD values of each group to evaluate the effects of different factors on cell proliferation and function. The optimal culture conditions were determined by analyzing the combination of different hormone concentrations and exposure times to maximize cell viability.

### 2.6 Effects of HYD and EGF on lactation-related physiological functions of equine mammary epithelial cells

#### 2.6.1 Cell culture

Mammary epithelial cells (EMECs) were seeded in culture medium containing 15% fetal bovine serum (FBS) at a density of 1 × 105 cells/mL. Cells were cultured in T25 flasks and six-well plates, with the T25 flasks designated for the measurement of triglycerides, lactose, total protein, and β-casein levels, and the six-well plates used for Oil Red O staining. After 24 h of incubation, hormone treatments were applied. The control group was maintained in medium containing 15% FBS, while the experimental group was treated with the optimal concentrations of hydrocortisone (HYD) and epidermal growth factor (EGF), and cultured for an additional 72 h.

#### 2.6.2 Cell collection and analysis

At the end of the culture period, the extracellular medium was first collected for β-casein content measurement. After removing the medium, the cells were washed twice with 1% PBS. Then, the cells were digested with 0.25% trypsin-EDTA. Following digestion, the cells were centrifuged at 1,000 × g, and the supernatant was discarded. The pellet was resuspended in 1 mL of 1% PBS and subjected to sonication for cell lysis to extract intracellular β-casein. The extracted cell samples were used for subsequent β-casein content analysis, which was quantified using an enzyme-linked immunosorbent assay (ELISA) kit (Shanghai Enzyme-Linked Biotechnology Co., Ltd., Catalog No. YJ423562). This method utilizes antibody binding to β-casein, triggering a colorimetric reaction. The optical density (OD) value was measured and used to calculate β-casein content.

For the measurement of triglycerides, lactose, and total protein, cells were similarly washed twice with 1% PBS to remove residual culture medium. The cells were then digested with 0.25% trypsin-EDTA, and after digestion, the cells were collected and prepared for subsequent analysis.

(1) Triglyceride (TG) Content Measurement: TG content was determined using an enzymatic assay kit (Nanjing Jiancheng Bioengineering Institute, Catalog No. A110-1-1). This method utilizes the hydrolysis of TG to release glycerol and fatty acids, and the absorbance is measured colorimetrically to calculate TG concentration.
(2) Lactose Content Measurement: Lactose content was measured using a colorimetric assay kit (Shanghai Enzyme-Linked Biotechnology Co., Ltd., Catalog No. ml077212). The reaction between lactose and the reagent results in a color change, and the concentration of lactose was calculated by measuring absorbance.
(3) Total Protein Content Measurement: Total protein content was determined using a colorimetric assay kit (Nanjing Jiancheng Bioengineering Institute, Catalog No. A045-3). Protein concentration was calculated by measuring absorbance at a specific wavelength and generating a standard curve.

Oil Red O Staining was used to assess lipid accumulation in the cells. After culture, the cells were stained using an Oil Red O staining kit (Beijing Solarbio Science & Technology Co., Ltd., Catalog No. G1262). Following staining, lipid accumulation was observed and documented using an inverted microscope.

### 2.7 Transcriptomics-based analysis of the effects of HYD and EGF on physiological functions related to lactation in equine mammary epithelial cells RNA quality inspection

#### 2.7.1 Cell culture and sample collection

In accordance with the cell culture procedures from the experiment “Effects of HYD and EGF on lactation-related physiological functions of equine mammary epithelial cells,” equine mammary epithelial cells (EMECs) were cultured in T25 flasks with 15% FBS medium for 24 h. After this, the experimental group was treated with hydrocortisone (HYD) and epidermal growth factor (EGF), and further cultured for 72 h. At the end of the culture period, the medium was discarded, and cells were collected using a cell scraper into 2 mL cryovials. The collected cells were immediately frozen in liquid nitrogen and sent to Novizen Biotechnology (Beijing) for transcriptome sequencing.

#### 2.7.2 RNA quality assessment

**Figure 1:**
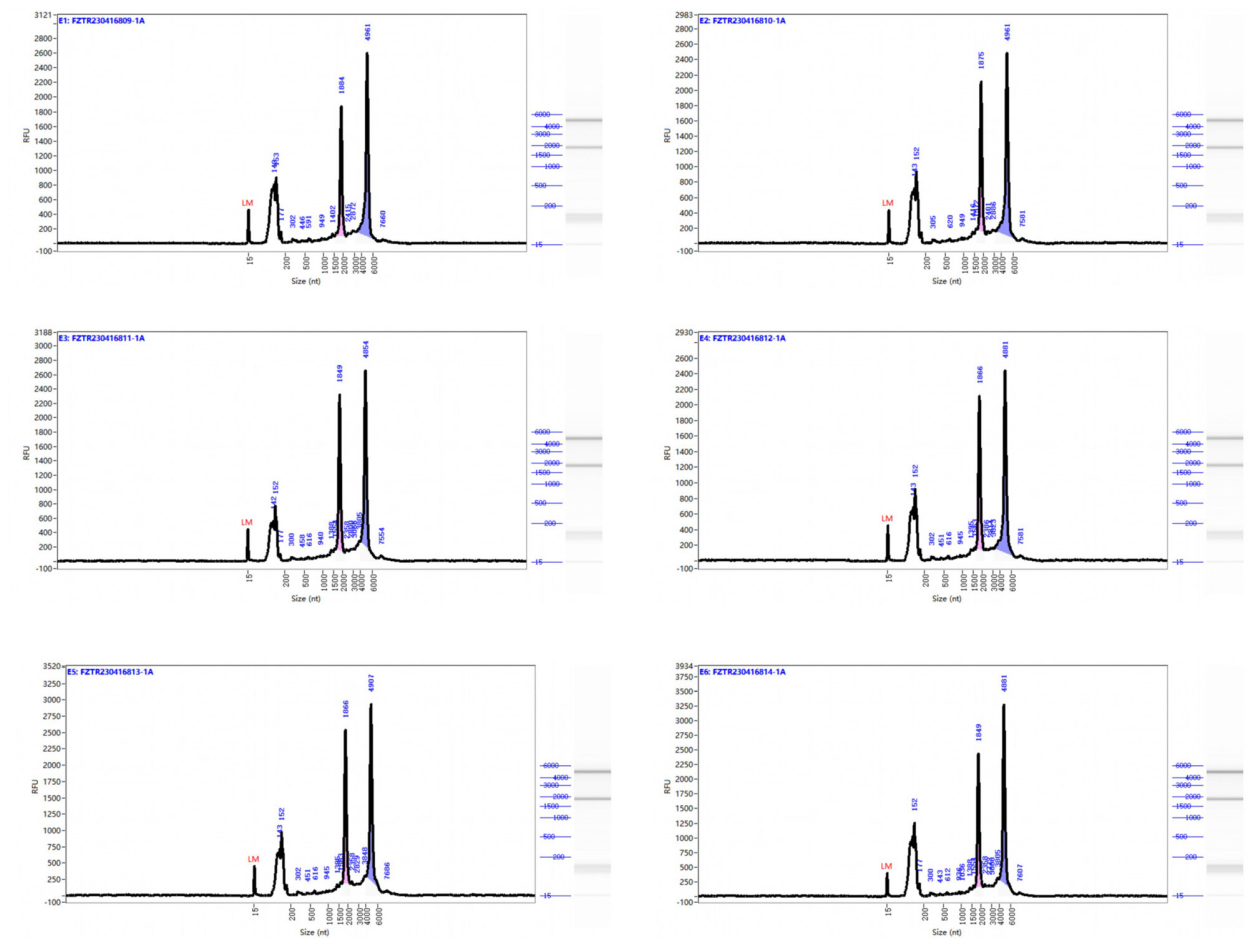
Capillary electrophoresis profiles and rna fragment distribution analysis of equine mammary epithelial cell samples. Size (nt): Represents the fragment distribution of the capillary sample. Fluorescence unit (FU): Refers to the real-time fluorescence signal intensity of the sample during capillary separation. A higher FU value indicates a higher sample concentration. Lower Marker: Refers to the reference material (non-sample fragments) analyzed alongside the sample, used for calibrating the fragment size and concentration of the sample. The right side shows the simulated gel image and fragment distribution.

RNA was extracted from EMECs, and RNA concentration was measured using Nanodrop. RNA integrity and purity were evaluated using the Agilent 2100 system. As shown in Table 5, RNA concentrations ranged from 92 to 140 ng/μL, with all RNA integrity numbers (RIN) greater than 7. The results were deemed “qualified,” indicating that the RNA samples were suitable for RNA library construction. Capillary electrophoresis and RNA fragment distribution analysis (see Figure 1) showed that the sample concentrations were above the reference markers, with no signs of degradation. The smooth baseline further confirmed the absence of contamination.

**Table 5:** Results of quality assays for RNA concentration in equine mammary epithelial cell samples.

**Sample name**	**Concentration ng/uL**	**RIN value**	**Detection conclusion**
C-1	93.00	7.70	Pass
C-2	104.00	7.90	Pass
C-3	92.00	8.10	Pass
T-1	99.00	8.00	Pass
T-2	107.00	8.10	Pass
T-3	140.00	7.60	Pass

C-1, C-2, C-3 represent the three samples of the control group; T-1, T-2, T-3 represent the three samples of the experimental group (HYD and EGF combined treatment group). The same applies to the table below. Pass: Sample quality meets library sequencing requirements.

#### 2.7.3 RNA extraction, library construction, and transcriptome data analysis

RNA samples were extracted using standard methods and library construction was performed using the NEBNext^®^ Ultra™ II RNA Library Prep Kit for Illumina, selected for its high efficiency in preparing RNA libraries for Illumina sequencing. The library was sequenced on the Illumina NovaSeq 6000 platform.

The sequencing results were aligned to the reference genome of *Equus caballus* using HISAT2, a rapid and precise RNA-seq data aligner. Gene expression levels were quantified using the FeatureCounts tool in Subread software, and gene expression data were filtered using DESeq2 (v1.20.0). Differentially expressed genes (DEGs) were identified using the thresholds of |log_2_(FoldChange)| ≥ 1 and *P* adj ≤ 0.05. Gene Ontology (GO) functional enrichment and Kyoto Encyclopedia of Genes and Genomes (KEGG) pathway enrichment analyses were performed using ClusterProfiler software, with a significance threshold of *P* adj < 0.05.

### 2.8 Statistical analysis

The obtained data were first pre-processed using Excel 2019 and analyzed with SPSS 18.0 to compare the results of the optimization test of EMEC culture conditions using One-way Analysis of Variance (ANOVA). The effects of hormones on the physiological function of the cells were evaluated using an independent samples *t*-test. A significance level of *P* < 0.05 was considered statistically significant. Graphs were created using GraphPad Prism 8.0.2.

The differential expression genes (DEGs) were selected using DESeq2 (v1.20.0). The data were first normalized using DESeq2, and DEGs were identified based on the criteria of |log_2_(FoldChange)| ≥ 1 and *P* adj ≤ 0.05. *P*-values were adjusted using the Benjamini–Hochberg method to control the false discovery rate. Gene Ontology (GO) functional enrichment analysis and KEGG pathway enrichment analysis were performed using ClusterProfiler software, with a significance threshold set at *P* adj < 0.05.

## 3 Results

### 3.1 Effect of adding different concentrations of serum and hormones alone on the viability of equine mammary epithelial cells

**Figure 2:**
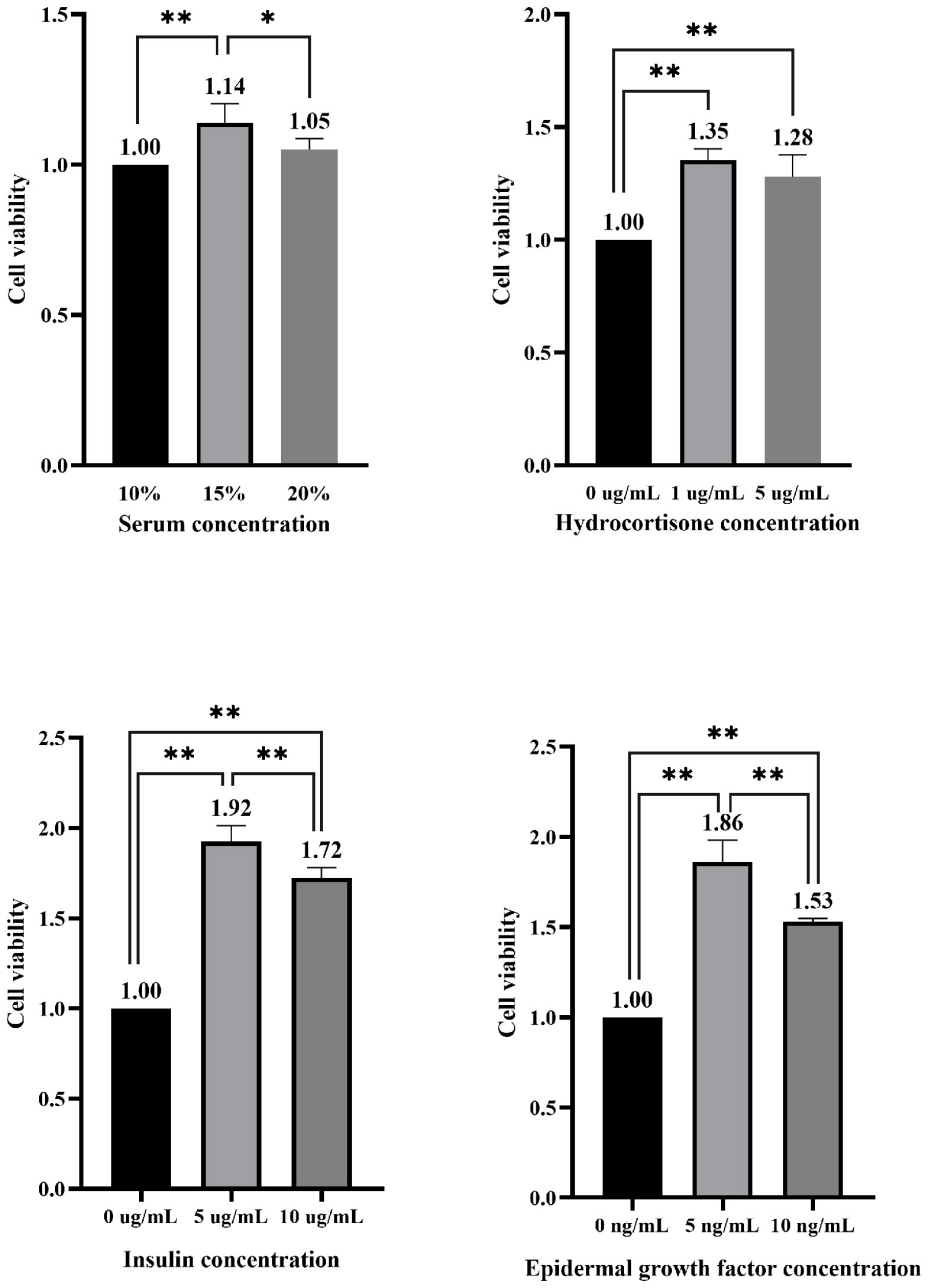
Effect of different concentrations of serum and hormones added alone on the viability of equine mammary epithelial cells. * and ** indicate statistically significant differences among groups at *p* < 0.05 and *p* < 0.01, respectively.

As shown in Figure 2, under the condition of 15% FBS, the viability of EMECs significantly increased by 14.00% (*P* < 0.01) compared to the 10% FBS concentration, and by 8.57% (*P* < 0.05) compared to the 20% FBS concentration. With 15% FBS, the addition of 1 and 5 μg/mL of HYD significantly enhanced cell viability, with increases of 35.00% and 28.00%, respectively (*P* < 0.01), compared to the 0 μg/mL group. The addition of 5 and 10 μg/mL of INS also significantly boosted cell viability, with increases of 92.00% (*P* < 0.01) and 72.00% (*P* < 0.01), and the 5 μg/mL group showed an 11.63% increase (*P* < 0.01) over the 10 μg/mL group. For EGF, the addition of 5 and 10 ng/mL resulted in significant increases in cell viability, with the 5 ng/mL group showing an 86.00% (*P* < 0.01) and 53.00% (*P* < 0.01) higher viability compared to the 0 ng/mL group. Furthermore, the 5 ng/mL group exhibited a significant 21.57% (*P* < 0.01) higher viability compared to the 10 ng/mL group.

### 3.2 Effect of mixed addition of different concentrations of HYD, INS and EGF on the viability of equine mammary epithelial cells

As shown in Table 6, group IV (1 μg/mL HYD and 5 ng/mL EGF) yielded the highest mean cell viability (1.19) among the nine conditions tested. Although one-way ANOVA with *post-hoc* analysis revealed no statistically significant differences across groups (*p* > 0.05), this condition consistently exhibited a favorable trend. Range (R) analysis further indicated that HYD was the dominant factor influencing viability (R = 0.14), followed by INS (R = 0.05) and EGF (R = 0.03).

**Table 6:** Orthogonal test results.

**Items**	**HYD, μg/mL**	**INS, μg/mL**	**EGF, ng/mL**	**Cell viability**
Test group I	0	0	0	1.00 ± 0.00
Test group II	0	5	5	1.02 ± 0.20
Test group III	0	10	10	1.14 ± 0.26
Test group IV	1	0	5	1.19 ± 0.24
Test group V	1	5	10	1.06 ± 0.14
Test group VI	1	10	0	0.99 ± 0.24
Test group VII	5	0	10	0.92 ± 0.22
Test group VIII	5	5	0	1.05 ± 0.24
Test group IX	5	10	5	0.84 ± 0.25
K1	1.05	1.03	1.01	
K2	1.08	1.04	1.02	
K3	0.94	0.99	1.04	
R	0.14	0.05	0.03	

K1, K2, K3: These values represent the average response values (cell viability) of different factors (such as HYD, INS, EGF) at their respective levels.

R: Represents the range of responses. R is the difference between the response values (K1, K2, K3) of each factor (such as HYD, INS, EGF). A larger R value indicates a greater influence of that factor on cell viability.

The experimental results are expressed as mean ± standard deviation.

### 3.3 Effects of HYD and EGF on lactation-related physiological functions of horse mammary epithelial cells

**Figure 3:**
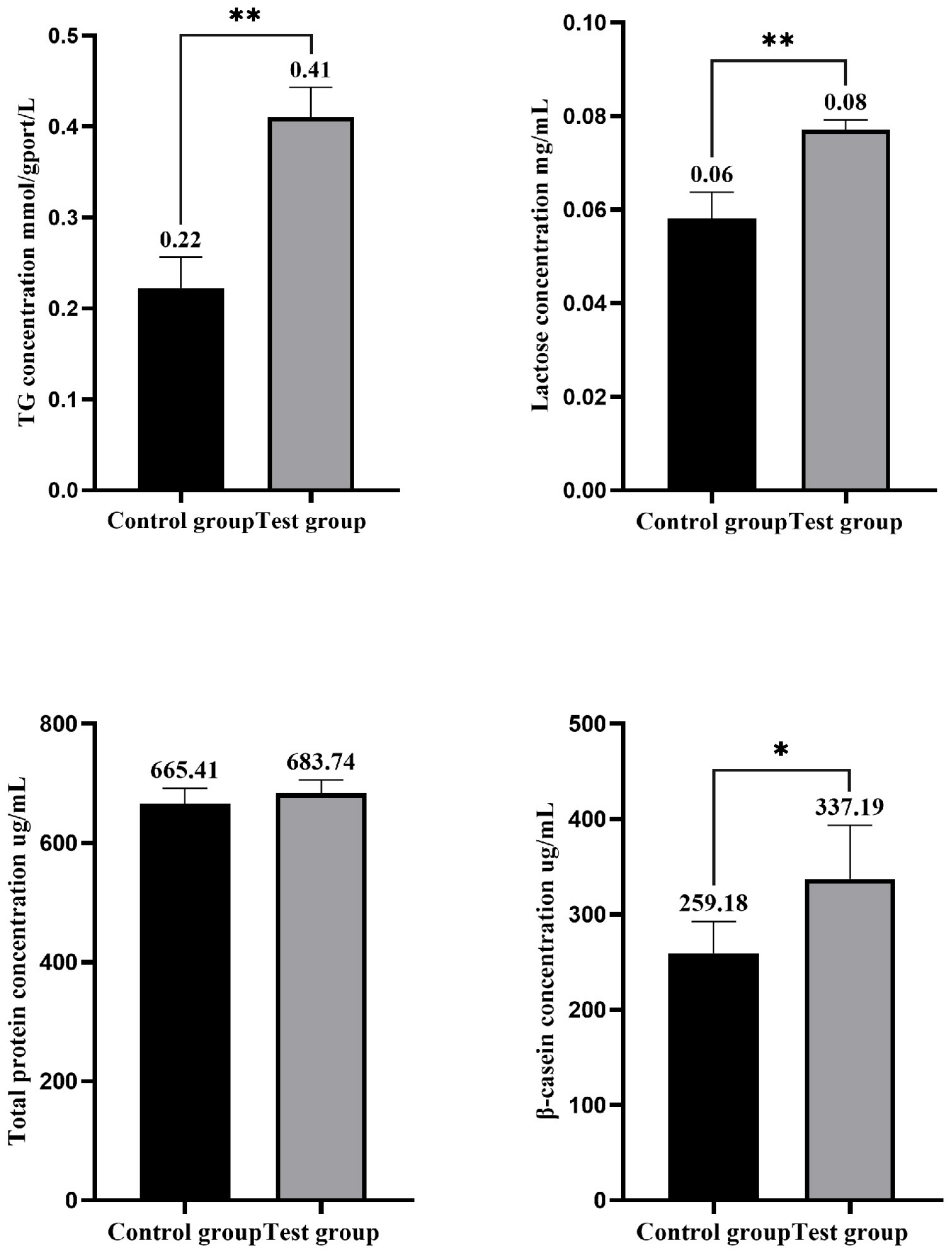
Effects of HYD and EGF on the physiological function of equine mammary epithelial cells. Control group represent the three samples of the control group; Test group represent the three samples of the experimental group (HYD and EGF combined treatment group). * and ** indicate statistically significant differences among groups at *p* < 0.05 and *p* < 0.01, respectively.

**Figure 4:**
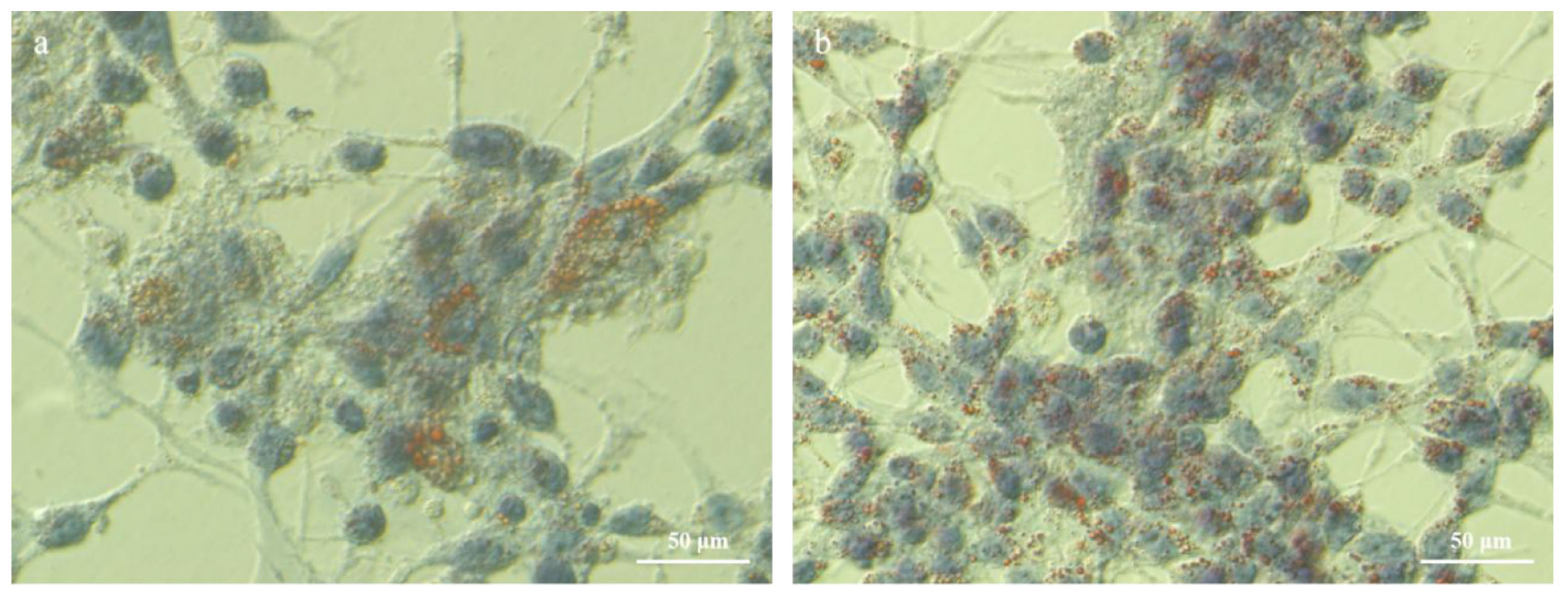
Effect of HYD and EGF on lipid droplet synthesis in equine mammary epithelial cells (400 × ). **(a)** Control group; **(b)** Test group(HYD+EGF group).

As presented in Figure 3, the TG concentration in the experimental group, following the addition of HYD and EGF, was significantly higher than in the control group, with an increase of 86.36% (*P* < 0.01). Similarly, the lactose concentration was significantly elevated, showing an increase of 33.33% (*P* < 0.01) compared to the control group. The total protein concentration increased by 2.75% compared to the control group, but this difference was not statistically significant (*P* > 0.05). The β-casein concentration in the experimental group was significantly higher, with an increase of 30.10% over the control group (*P* < 0.05). Figure 4 illustrates an increase in lipid droplet content in the experimental group, suggesting that HYD and EGF promote lipid droplet synthesis in EMECs.

### 3.4 RNA-Seq data quality assessment

To ensure the accuracy and reliability of transcriptomic data, we performed quality control analysis on RNA-Seq data obtained from six equine mammary epithelial cell samples. The number of raw reads ranged from approximately 39.5 million to 61.1 million across samples, with clean reads accounting for over 95% of the total, indicating high sequencing quality. The sequencing error rate was consistently below 0.03% for all samples. Additionally, the proportion of bases with Phred scores above 20 (Q20) and 30 (Q30) remained above 97% and 93%, respectively, reflecting high base-calling accuracy. GC content ranged from 53.12% to 56.13%, which is consistent with the typical GC composition of equine mRNA and suggests no significant GC bias. The data quality control results are detailed in Table 7.

**Table 7:** Quality control of RNA-Seq data from equine mammary epithelial cell samples.

**Sample**	**Raw reads**	**Clean reads**	**Error rate (%)**	**Q20 (%)**	**Q30 (%)**	**GC (%)**
C-1	49, 047, 412	47, 453, 936	0.03	97.49	93.75	54.66
C-2	47, 225, 696	44, 589, 760	0.02	97.84	94.48	55.02
C-3	39, 533, 378	38, 094, 966	0.03	97.75	94.28	53.12
T-1	61, 136, 730	58, 385, 046	0.03	97.30	93.24	54.42
T-2	48, 037, 914	45, 964, 542	0.03	97.38	93.57	54.47
T-3	50, 199, 146	47, 551, 068	0.03	97.26	93.31	56.13

Sample, name of the sample; Raw reads, number of reads in the raw data; Clean reads, number of reads in the raw data filtered; Error rate, overall sequencing error rate of the data; Q20, Percentage of total bases with Phred value greater than 20; Q30, Percentage of total bases with Phred value greater than 30; GC, Percentage of G and C in Clean reads.

### 3.5 Reference genome matching

To assess the alignment of the EMEC samples with the reference genome, the sequencing data were compared to the Equus caballus (horse) genome. The alignment results, as shown in Table 8, indicated that the total alignment for each sample exceeded 93%, with the highest alignment being 94.45% and the lowest 93.39%. The unique mapping rates were above 89%, with the highest at 91.20% and the lowest at 89.94%, suggesting a high level of accuracy in the alignment, which was suitable for further analysis.

**Table 8:** Reference genome comparison results.

**Sample**	**Total map (%)**	**Unique map (%)**	**Multi map (%)**	**Read1 map (%)**	**Read2 map (%)**
C-1	94.11	90.74	3.37	45.42	45.32
C-2	94.45	91.20	3.24	45.60	45.61
C-3	94.16	90.6	3.55	45.30	45.30
T-1	93.39	89.94	3.45	45.15	44.79
T-2	93.48	90.11	3.37	45.15	44.96
T-3	93.79	90.36	3.43	45.38	44.98

Sample: the name of the sample; Total map: the number and percentage of reads matched to the genome; Unique map: the number and percentage of reads matched to a unique position in the reference genome (used for subsequent quantitative data analysis of reads); Multi map: the number and percentage of reads matched to multiple positions in the reference genome; read1 map: number and percentage of reads aligned to the reference genome; read2 map: number and percentage of reads aligned to the reference genome.

### 3.6 Quantitative analysis of gene expression

**Figure 5:**
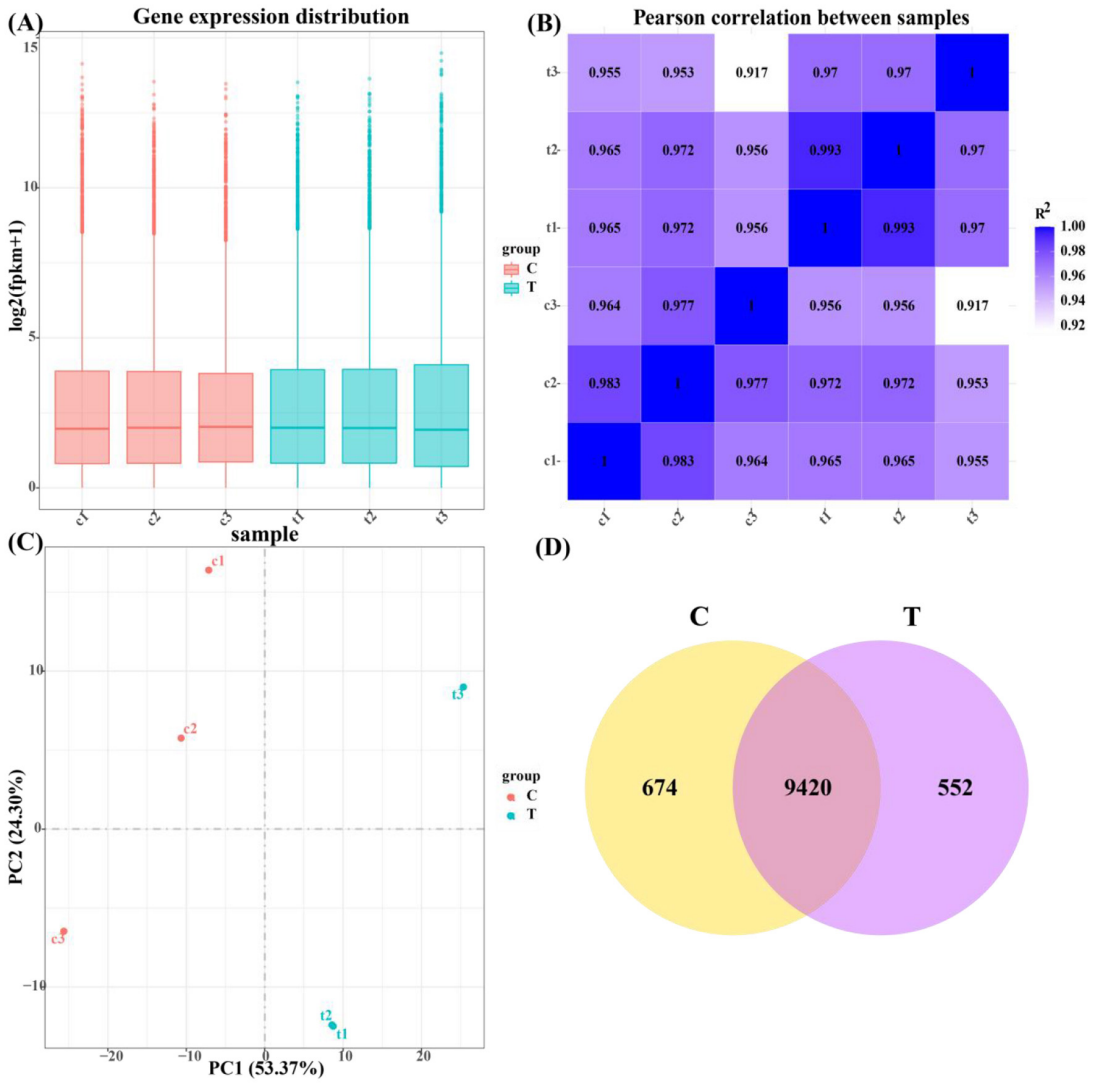
Quantitative analysis of gene expression. **(A)** Box plot of gene expression distribution of samples. **(B)** Heat map of inter-sample correlation. **(C)** Plot of results of principal component analysis. **(D)** Coexpression of a Venn diagram.

As illustrated in Figure 5A, the gene expression levels within the control and test groups exhibited minimal variation, demonstrating the reproducibility of the samples for subsequent research analysis. This consistency suggests that the samples are reliable and that any observed differences in gene expression are likely attributable to the experimental conditions rather than sample variability.

As presented in Figure 5B, the Pearson correlation coefficients between duplicate samples within both the control and experimental groups were greater than 0.917, indicating a strong correlation. The Pearson correlation coefficient is a measure of the linear relationship between two sets of data, and a high value confirms that the replicates of each group exhibit similar expression profiles, reinforcing the reliability and reproducibility of the data for further analysis.

Figure 5C shows principal component analysis (PCA), where PC1 and PC2 contributed 53.37% and 24.30%, respectively, to the variance in the dataset. PCA is a dimensionality reduction technique used to summarize the variability in gene expression data by projecting it onto principal components. The clear distinction observed between the control and experimental groups along PC1 indicates that the hormone treatment induced a significant shift in gene expression, highlighting the impact of the experimental conditions on the overall transcriptional profile.

As presented in Figure 5D, 674 genes were identified as expressed in the control group, 552 genes in the test group, and 9,420 genes were expressed in both groups. This analysis provides insight into the gene expression overlap and identifies differentially expressed genes between the control and test conditions, which can be further explored for their roles in the studied biological processes.

### 3.7 Differential gene expression and enrichment analysis

**Figure 6:**
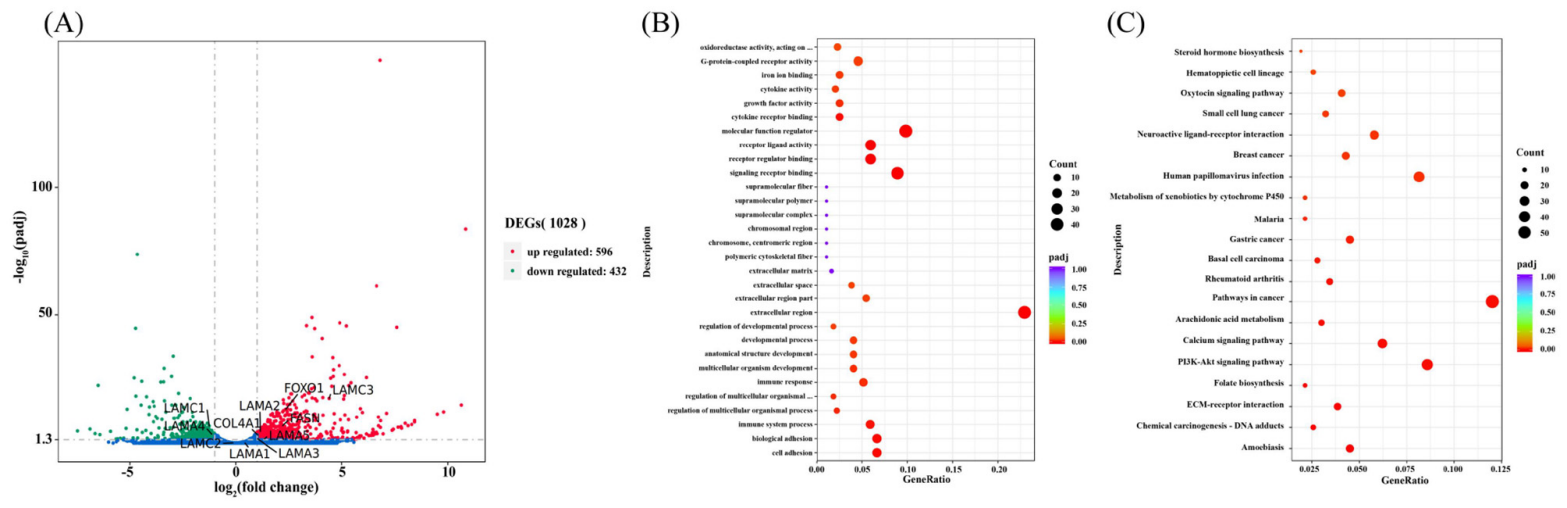
Differential gene expression and enrichment analysis. **(A)** Differential gene expression analysis. **(B)** GO function enrichment analysis. **(C)** KEGG pathway enrichment analysis.

To assess the transcriptional impact of hormone treatment (HYD + EGF) on equine mammary epithelial cells (EMECs), differential gene expression analysis was performed. As shown in Figure 6A, a volcano plot visualized the distribution of all detected transcripts, with log2 (Fold Change) on the x-axis and –log10(padj) on the y-axis. Among the 19,786 genes detected, 596 were significantly upregulated and 432 were downregulated based on the criteria of padj < 0.05 and |log2 FC| ≥ 1. The upregulated genes are located on the right-hand side of the plot, indicating induction by hormone treatment, while the downregulated genes appear on the left side, suggesting suppression. This pattern suggests a distinct transcriptional response of EMECs to the combined hormonal stimulation.

From the volcano plot, several genes, including laminin subunit alpha-2 (LAMA2), laminin subunit alpha-3 (LAMA3), laminin subunit alpha-4 (LAMA4), laminin subunit alpha-5 (LAMA5), laminin subunit gamma-1 (LAMC1), laminin subunit gamma-3 (LAMC3), type IV collagen alpha-1 chain (COL4A1), fatty acid synthase (FASN), and forkhead box protein O1 (FOXO1), were significantly expressed. These genes are likely involved in mare lactation and milk component synthesis, suggesting their potential role in the hormonal regulation of mammary gland function.

### 3.8 GO function enrichment analysis

To further explore the biological significance of the differentially expressed genes, Gene Ontology enrichment analysis was conducted across three categories: biological process, molecular function, and cellular component. As illustrated in Figure 6B, DEGs were significantly enriched in biological processes such as cell adhesion, biological adhesion, immune response, and developmental process. Molecular functions included cytokine activity, growth factor activity, receptor binding, and signaling receptor activity. Enrichment in cellular components highlighted regions such as the extracellular matrix, extracellular region, and supramolecular complexes. These enriched terms suggest that hormone treatment affects key physiological processes in EMECs including immune regulation, cell signaling, and structural organization.

### 3.9 KEGG pathway enrichment analysis

To explore the impact of hormones on the signaling pathways associated with the physiological functions of EMECs, KEGG pathway enrichment analysis was performed on the differentially expressed genes. A total of 23 pathways were identified as enriched, as shown in Figure 6C, the DEGs were significantly enriched in pathways including ECM–receptor interaction, PI3K–Akt signaling, calcium signaling, and arachidonic acid metabolism. Notably, the ECM–receptor interaction and PI3K–Akt pathways are closely related to cell proliferation, adhesion, and lipid biosynthesis, all of which are critical to mammary gland function and lactation. These findings support the hypothesis that HYD and EGF regulate EMEC physiological functions through structural remodeling and metabolic reprogramming via these signaling cascades.

## 4 Discussion

### 4.1 Optimization of culture conditions of equine mammary epithelial cells

MEC viability plays a pivotal role in regulating mammary metabolism, and optimizing cell culture conditions is essential for enhancing cell viability and proliferation efficiency, thereby improving milk composition. The culture medium serves as the foundation for *in vitro* cell growth, with FBS being the most commonly used supplement. It contains a wide array of essential nutrients, including proteins, peptides, and growth regulators, which create a conducive environment for cell proliferation, differentiation, and the maintenance of cell morphology and function (12). However, FBS is complex in composition, and while adding a certain percentage of FBS to the culture medium promotes cell growth and division, excessively high concentrations can hinder growth or even lead to cell death. Kwon et al. (13) demonstrated that adding varying concentrations of FBS (0%-100%) to cultures of cow MECs caused a gradual increase in cell proliferation at concentrations ranging from 0% to 20%. However, at concentrations between 25% and 100%, cell proliferation rates started to decrease. Similarly, Sattari et al. (14) observed that when 10%, 5%, 1.25%, and 0% FBS were added to dairy MEC cultures, the growth rate at 5% FBS was comparable to that at 10%, while 0% FBS resulted in the lowest growth rate. In this study, the addition of 10%, 15%, and 20% FBS to EMEC cultures revealed that the optimal cell viability was achieved at 15% FBS concentration. FBS provides essential nutrients for cell growth, and when the concentration is too low, cell growth factors become limited, leading to decreased viability. However, when the serum concentration is too high, endotoxin levels in the serum increase, which may explain the reduced cell viability at 20% FBS concentration (15).

HYD is a key endogenous hormone involved in mammary gland alveolar development in female animals. It is known for its anti-inflammatory properties, its ability to maintain stable cell morphology, improve cell adhesion, and inhibit fibroblast growth (16). Gaffney and Pigott (17) found that adding 5 μg/mL of HYD to human MEC cultures promoted rapid cell proliferation. In this study, varying concentrations of HYD (0, 1, and 5 μg/mL) were added to the culture medium of EMECs. All concentrations enhanced cell viability, with the highest viability observed at 1 μg/mL. However, cell viability decreased at 5 μg/mL, likely due to a reduction in the expression of the HYD receptor at higher concentrations.

EGF is known to promote rapid cell growth and differentiation while mitigating mechanical cell damage (18–20). Sobolewska et al. (21) demonstrated that the addition of EGF to the culture medium activates the mTOR signaling pathway, thereby promoting the proliferation of MECs in dairy cows. In this experiment, 0, 5, and 10 ng/mL of EGF were added to the culture medium, and the results revealed that cell viability peaked at 5 ng/mL. However, viability decreased at 10 ng/mL. This reduction in cell viability may be attributed to the high EGF concentration, which inhibits the expression of signal transducer and activator of transcription 5 (STAT5) and disrupts glucose metabolism (22).

INS is a key regulator of cell proliferation, functioning similarly to EGF. Upon binding to the INS receptor, it induces DNA synthesis in cells, stimulating rapid cell division and proliferation (23). Zhao et al. (24) demonstrated that treatment with varying concentrations of INS (0, 5, 50, and 500 ng/mL) promoted cell proliferation in MECs from dairy cows. In this study, the highest cell viability was observed at 5 μg/mL of INS, whereas cell viability decreased at 10 μg/mL. This decrease may be attributed to insufficient INS receptor expression (25). Additionally, Bionaz and Loor (26) found that the addition of INS to dairy cow MEC cultures resulted in a significant negative correlation with Janus kinase 2 (JAK2) expression as INS concentration increased. Since STAT5, a substrate of JAK, plays a key role in cell proliferation and differentiation, the reduced expression of JAK2 and STAT5 at high INS concentrations may contribute to the observed decrease in cell viability at 10 μg/mL of INS in this study.

Mammary tissue is highly complex, with the proliferation and differentiation of MECs being critical factors in mammary gland development. Various hormones interact during this process, making it essential to evaluate their combined effects on MECs. Identifying the optimal combination of hormones and their concentrations is key to ensuring ideal lactation conditions, promoting mammary tissue development, and enhancing milk composition. In this study, an L9 (11) orthogonal experimental design was employed, where different concentrations of HYD, INS, and EGF were added to assess the effects of these combined hormones on EMEC viability. The results demonstrated that cell viability was highest when the HYD concentration was 1 μg/mL and the EGF concentration was 5 ng/mL. Among the three hormones, the order of their effects on cell viability was HYD > INS > EGF, indicating that HYD had the most significant impact on cell viability. Furthermore, when the hormones were combined, the best cell viability was observed at an INS concentration of 0 μg/mL, with the lowest viability at 10 μg/mL. This decline in cell viability at higher INS concentrations may be due to a reduction in JAK2 and STAT5 expression, which impairs glucose metabolism and inhibits cell proliferation. Additionally, Qiu et al. (27) found that HYD antagonizes INS by inhibiting the binding of INS to its receptor and regulating glucose transport on the INS receptor, which could also explain the results observed in this experiment.

### 4.2 Effects of HYD and EGF on lactation-related physiological functions of equine mammary epithelial cells

Milk fat, primarily composed of TGs, plays a pivotal role in determining the flavor of milk and providing essential nutrients and energy to the organism. Collier et al. (28) demonstrated that the addition of HYD to the MEC culture medium of dairy cows resulted in an increased fatty acid content. Forsyth and Turvey (29) showed that adding HYD to the MEC culture medium of goats significantly enhanced the milk fat fatty acid content synthesized from scratch. In the present study, after adding HYD and EGF to the culture medium of EMECs, a significant increase in lipid droplet content was observed through oil red O staining, along with a highly significant increase in TG content. These findings are consistent with previous studies. The observed effects may be attributed to the promotion of acetyl-CoA carboxylase (ACC) and fatty acid synthase (FASN) gene expression by HYD. Both ACC and FASN are key enzymes in fatty acid biosynthesis; FASN catalyzes the conversion of acetyl-CoA into malonyl-CoA, a precursor that ultimately leads to the synthesis of TG and phospholipids (30).

Lactose is primarily produced in the Golgi apparatus of MECs, where glucose is transported into the cell via the glucose transporter protein (GLUT) and then converted to galactose by a series of catalytic enzymes such as hexokinase (HKS). Finally, lactose synthase catalyzes the formation of lactose. Casey and Plaut (4). demonstrated that HYD can restore lactose synthase activity and promote lactose synthesis. Haney (31). found that the addition of HYD to mouse MECs increased the expression of glucose transporter protein 8 (GLUT8), which regulates glucose uptake and subsequently enhances lactose content. Li (32). showed that the addition of EGF to the culture medium of dairy cow MECs significantly increased the expression of the lactose synthesis-related gene GLUT1, though this effect was suppressed as EGF concentration increased. In this study, the addition of HYD and EGF to EMEC cultures resulted in a highly significant increase in lactose content, which may be attributed to the upregulation of key lactose synthesis genes, GLUT8 and GLUT1, thereby promoting glucose transport and lactose production.

Milk protein is a key component of milk, and its concentration plays a major role in determining milk quality. HYD is essential for milk protein accumulation, binding to specific receptors in the mammary gland to stimulate the expression of milk protein genes, thus regulating the secretion of β-casein (33). Manjarín et al. (34). demonstrated a positive correlation between the presence of the HYD-specific receptor and the expression of the CSN2 gene. Luetteke et al. (35) suggested that knockdown of the EGF receptor reduced the expression of milk protein synthesis genes. The results of the current study show that adding HYD and EGF to the culture medium of EMECs significantly increased β-casein content. This effect may be due to the binding of HYD and EGF to their respective receptors, which in turn promotes the expression of genes related to casein synthesis, enhancing casein secretion.

### 4.3 Transcriptomic-based analysis of the effects of HYD and EGF on lactation-related physiological functions of equine mammary epithelial cells

Gene expression is a key determinant of lactation performance in female livestock, and identifying lactation-associated genes is crucial for the regulation and genetic improvement of milk traits. In this study, EMECs were treated with hormones to investigate transcriptional responses. The results indicated upregulation of several laminin (LN) family genes and type IV collagen genes, which were enriched in the ECM-receptor interaction pathway. Additionally, lipid metabolism-related pathways, particularly the PI3K-Akt signaling cascade, were suggested to be activated with a marked increase in the expression of key metabolic genes such as FASN, implying that multiple signaling molecules may contribute to the regulation of lactation.

Specifically, LN subunit genes, including LAMA2, LAMA3, LAMA4, LAMA5, LAMC1, and LAMC3, which are closely associated with epithelial cell proliferation and differentiation, were upregulated under hormone stimulation and enriched in the ECM-receptor interaction pathway. GO and KEGG enrichment analyses highlighted their involvement in biological processes such as cell adhesion, biological adhesion, developmental processes, and receptor binding. LNs, as major structural components of the ECM, not only contribute to intercellular connectivity and the maintenance of cellular architecture but also act as intermediaries in signal transduction between hormones and target cells (11, 36, 37). Previous studies have demonstrated that LN can enhance cell adhesion, proliferation, and differentiation (38). Josan et al. (39) further reported that 3T3-L1 adipocytes cultured in LN-rich ECM exhibited elevated expression of SREBP1 and PPARγ, two pivotal regulators of lipid biosynthesis, suggesting that LN may regulate lipid metabolism via signal transduction mechanisms. In line with this, this study observed upregulation of COL4A1, a key constituent of type IV collagen, which, together with LN, forms the basement membrane and may play an important role in maintaining mammary gland architecture and function by modulating cell adhesion and signaling.

Lipid biosynthesis is a fundamental metabolic process in lactation, directly contributing to milk composition and lactation capacity. In this study, hormone treatment appeared to enhance lipid biosynthetic activity in EMECs, as evidenced by increased FASN expression, elevated TG levels, and more abundant lipid droplet formation. FASN, localized in the cytoplasm, catalyzes the synthesis of saturated fatty acids using acetyl-CoA, malonyl-CoA, and NADPH, and functions as a rate-limiting enzyme in TG synthesis. FASN is known to play a pivotal role in mammary gland development and lactation (40).

At the transcriptional level, FASN is primarily activated by SREBP1, a master transcription factor that regulates lipogenic gene expression. SREBP1 activity is positively regulated by the PI3K-Akt signaling pathway, while FOXO1 acts as a negative regulator by binding to the SREBP1 promoter and inhibiting its transcription (41, 42). In our dataset, hormone treatment was associated with reduced FOXO1 expression while FASN was upregulated, accompanied by increased TG content and lipid droplet accumulation, indicating a possible involvement of the PI3K-Akt pathway. Previous studies have shown that phosphorylated Akt enhances SREBP1 transcriptional activity, thereby promoting FASN expression. Concurrently, Akt-mediated phosphorylation of FOXO1 leads to its nuclear export and inactivation, relieving its inhibitory effect on SREBP1 (43).

Taken together, our findings suggest that hormonal stimulation may influence MECs by modulating both structural and metabolic pathways. The enrichment of LAMA family members and COL4A1 in ECM-receptor pathways highlights their potential role in maintaining microenvironmental stability, cell adhesion, and epithelial differentiation. Simultaneously, the observed changes in the PI3K-Akt/SREBP1-FASN axis and suppression of FOXO1 imply a coordinated mechanism that could promote milk fat synthesis. As an exploratory study, these results provide initial insights into the transcriptional regulation of lactation performance in mares and identify potential candidate genes for future functional validation and genetic improvement.

## 5 Conclusions

This study systematically evaluated the effects of hydrocortisone (HYD), insulin (INS), and epidermal growth factor (EGF) on the viability and lactation-related functions of equine mammary epithelial cells. Through single-factor screening, the concentrations of each hormone were determined, and an orthogonal experimental design was subsequently applied to examine their combined application. The results suggested that the combination of HYD and EGF may enhance cell viability and support the synthesis of milk components, including triglycerides, lactose, and β-casein.

Transcriptomic analysis further revealed that hormone treatment was associated with upregulation of genes involved in cellular structure maintenance and lactation. In particular, members of the laminin gene family (LAMA2, LAMA3, LAMA5, LAMC1) and COL4A1 were enriched in the ECM–receptor interaction pathway, suggesting their potential role in maintaining cell adhesion and epithelial integrity. In addition, lipid metabolism–related genes such as FASN and its upstream transcription factor SREBP1 were upregulated, while FOXO1 was downregulated, implying possible involvement of the PI3K–Akt signaling pathway in hormone-induced regulation of milk fat synthesis.

In summary, this exploratory study highlights the potential influence of HYD and EGF in regulating lactation-related functions in equine mammary epithelial cells. The identification of several key signaling pathways and candidate genes provides preliminary insights into the transcriptional regulation of lactation in mares and offers a valuable foundation for future functional validation and applied research aimed at improving milk quality.

## Data Availability

The data presented in this study are publicly available. This data can be found here: https://www.ncbi.nlm.nih.gov, accession number PRJNA1198216.
