# Supplementary material for: Effects of added exogenous hormones on lactation-related physiological functions of equine mammary epithelial cells
Source: Front Vet Sci. 2025 Nov 18;12:1660502. doi: 10.3389/fvets.2025.1660502 (PMC12671389; doi:10.3389/fvets.2025.1660502)
Supplement: Supplementary file 1 [file Supplementary_file_1.docx]

Attachment 1:

**Solution Preparation Methods**

1% PBS: Add 500 μL of penicillin-streptomycin to 50 mL of PBS, store at 4°C.

2% PBS: Add 1 mL of penicillin-streptomycin to 50 mL of PBS, store at 4°C.

Cell Freezing Medium: DMEM/F12 medium:FBS:DMSO = 7:2:1, prepare fresh as needed.

0.1% Type I Collagenase: Dissolve 25 mg of Type I collagenase in 25 mL of PBS, filter through a 0.22 μm filter, prepare fresh as needed.

0.01% Hyaluronidase: Dissolve 2.5 mg of hyaluronidase in 25 mL of PBS, filter through a 0.22 μm filter, prepare fresh as needed.

10% Serum Medium: Add 500 μL of penicillin-streptomycin and 5 mL of fetal bovine serum (FBS) to 50 mL of DMEM/F12 medium, filter through a 0.22 μm filter, store at 4°C.

15% Serum Medium: Add 500 μL of penicillin-streptomycin and 7.5 mL of fetal bovine serum (FBS) to 50 mL of DMEM/F12 medium, filter through a 0.22 μm filter, store at 4°C.

20% Serum Medium: Add 500 μL of penicillin-streptomycin and 10 mL of fetal bovine serum (FBS) to 50 mL of DMEM/F12 medium, filter through a 0.22 μm filter, store at 4°C.

Hydrocortisone Stock Solution Preparation: Dissolve 20 mg of hydrocortisone in 20 mL of anhydrous ethanol, yielding a stock concentration of 1 mg/mL, filter through a 0.22 μm filter, store at -20°C.

Insulin Stock Solution Preparation: Accurately measure 83.33 μL of concentrated HCl, dilute to 100 mL to achieve a concentration of 0.01 mmol/L; dissolve 20 mg of insulin in 8 mL of 0.01 mmol/L HCl, yielding a stock concentration of 2.5 mg/mL, filter through a 0.22 μm filter, store at -20°C.

Epidermal Growth Factor (EGF) Stock Solution Preparation: Dissolve 20 μg of epidermal growth factor in 10 mL of PBS, yielding a stock concentration of 2 μg/mL, filter through a 0.22 μm filter, store at -20°C.
